# The MEK-Inhibitor Selumetinib Attenuates Tumor Growth and Reduces IL-6 Expression but Does Not Protect against Muscle Wasting in Lewis Lung Cancer Cachexia

**DOI:** 10.3389/fphys.2016.00682

**Published:** 2017-01-18

**Authors:** Ernie D. Au, Aditya P. Desai, Leonidas G. Koniaris, Teresa A. Zimmers

**Affiliations:** ^1^Department of Surgery, Indiana University School of MedicineIndianapolis, IN, USA; ^2^Department of Biochemistry and Molecular Biology, Indiana University School of MedicineIndianapolis, IN, USA; ^3^Indiana University Simon Cancer CenterIndianapolis, IN, USA; ^4^IUPUI Center for Cachexia Research, Innovation and TherapyIndianapolis, IN, USA; ^5^Department of Otolaryngology, Head and Neck Surgery, Indiana University School of MedicineIndianapolis, IN, USA; ^6^Department of Anatomy and Cell Biology, Indiana University School of MedicineIndianapolis, IN, USA

**Keywords:** cachexia, atrophy, cancer, cytokines, chemotherapy, Interleukin-6, MAP Kinase, lung neoplasms

## Abstract

Cachexia, or wasting of skeletal muscle and fat, afflicts many patients with chronic diseases including cancer, organ failure, and AIDS. Muscle wasting reduces quality of life and decreases response to therapy. Cachexia is caused partly by elevated inflammatory cytokines, including interleukin-6 (IL-6). Others and we have shown that IL-6 alone is sufficient to induce cachexia both *in vitro* and *in vivo*. The mitogen-activated protein/extracellular signal-regulated kinase kinase (MEK) inhibitor Selumetinib has been tested in clinical trials for various cancers. Moreover, Selumetinib has also been shown to inhibit the production of IL-6. In a retrospective analysis of a phase II clinical trial in advanced cholangiocarcinoma, patients treated with Selumetinib experienced significant gains in skeletal muscle vs. patients receiving standard therapy. However, the use of Selumetinib as a treatment for cachexia has yet to be investigated mechanistically. We sought to determine whether MEK inhibition could protect against cancer-induced cachexia in mice. *In vitro*, Selumetinib induced C2C12 myotube hypertrophy and nuclear accretion. Next we tested Selumetinib in the Lewis lung carcinoma (LLC) model of cancer cachexia. Treatment with Selumetinib reduced tumor mass and reduced circulating and tumor IL-6; however MEK inhibition did not preserve muscle mass. Similar wasting was seen in limb muscles of Selumetinib and vehicle-treated LLC mice, while greater fat and carcass weight loss was observed with Selumetinib treatment. As well, Selumetinib did not block wasting in C2C12 myotubes treated with LLC serum. Taken together, out results suggest that this MEK inhibitor is not protective in LLC cancer cachexia despite lowering IL-6 levels, and further that it might exacerbate tumor-induced weight loss. Differences from other studies might be disease, species or model-specific.

## Introduction

Cachexia is a devastating consequence of cancer and other chronic diseases recognized by dysmetabolism leading to a progressive reduction in skeletal muscle and adipose tissue (Fearon et al., 2011; Argilés et al., 2015; Tsoli et al., 2016). Muscle wasting reduces function, quality of life and decreases response to therapy. Low muscle mass increases chemotherapy toxicity, while chemotherapy in turn can cause muscle wasting and contribute to cachexia (Chen et al., 2015; Barreto et al., 2016; de Lima Junior et al., 2016; Toledo et al., 2016). Currently there are no approved, effective therapies for cachexia. However, blocking muscle loss in cancer cachexia prolongs function and life, indicating that anti-cachexia therapies will be an essential adjunct to anti-tumor therapies for treatment of cancer (Benny Klimek et al., 2010; Zhou et al., 2010; Hatakeyama et al., 2016).

There are several underlying mechanisms that directly contribute to cachexia. It has been referred to as a syndrome of energy imbalance, where intake is decreased and expenditure is increased. However, even with a controlled energy intake, this imbalance persists (Evans et al., 1985). The loss of skeletal muscle mass is largely attributed to abnormalities in protein metabolism, where degradation outweighs synthesis caused in part by increased activity of the ubiquitin-proteasome pathway as well as autophagy (Acharyya and Guttridge, 2007; Mammucari et al., 2007). Loss of myofibrillar proteins leads directly to muscle atrophy, weakness, and fatigue. Common catabolic pathways involved in turnover of skeletal muscle proteins are induced by a multitude of inflammatory cytokines, both tumor- and host-derived. These cachectic mediators include TNFα, Myostatin, Activin, other members of the TGF-β superfamily, and the well-known driver of cachexia, Interleukin-6 (IL-6) (Jackman and Kandarian, 2004; Fearon et al., 2012; Tsoli and Robertson, 2013; Narsale and Carson, 2014; Londhe and Guttridge, 2015). IL-6 binds IL-6 receptor and the common signaling receptor GP130 to activate the ERK, AKT, and STAT3 pathways (Belizário et al., 2016). Others and we have shown that IL-6 alone is sufficient to induce muscle wasting both *in vitro* and *in vivo* (Bonetto et al., 2011, 2012; Zimmers et al., 2016), largely through activation of STAT3 (Zimmers et al., 2016) downstream of GP130 and JAK. Inhibition of IL-6, IL-6 receptor, or STAT3 all reduce cachexia in experimental systems (Strassmann et al., 1992; Oldenburg et al., 1993; White et al., 2011; Silva et al., 2015). Moreover, anti-IL-6 therapies have shown promise in human lung cancer cachexia (Bayliss et al., 2011).

In addition to IL-6, a variety of mitogenic and inflammatory stimuli can activate the Mitogen Activated Protein Kinase (MAPK)/ERK pathway, including other cytokines and growth factors signaling through tyrosine kinase receptors (Guan, 1994). MEK1/2 phosphorylates ERK and influences survival, growth, proliferation, and inflammatory processes (Zheng and Guan, 1993; Hommes et al., 2003). The MEK pathway is also activated by oncogenic Ras, and has been targeted for anti-cancer therapies (Neuzillet et al., 2014). The selective small molecule MEK1/2 inhibitor Selumetinib decreases phosphorylation and activation of ERK1/2 (Yeh et al., 2007) and shows efficacy in cancers of the lung, skin, ovary and liver (Miller et al., 2014; Facciorusso et al., 2015; Heigener et al., 2015; Shoushtari and Carvajal, 2016).

A Phase II study of Selumetinib showed weight gain in patients with bililary cancer, a condition typically associated with severe wasting (Bekaii-Saab et al., 2011). Retrospective re-analysis of those data showed that patients who received Selumetinib experienced significant gains in skeletal muscle while those on standard therapy experienced muscle loss (Bekaii-Saab et al., 2011; Prado et al., 2012). Inhibition of the ERK pathway, via a dominant negative form of Raf or a pharmacological inhibitor, results in robust myotube hypertrophy (Rommel et al., 1999). Additionally, ERK inhibition de-represses myogenic differentiation caused by cardiotrophin-1, a member of the IL-6 family of cytokines (Miyake et al., 2009). Pharmacological inhibition of ERK1/2 significantly increases mRNA levels of the transcription factor myogenin, promoting differentiation and expression of muscle specific genes and the myogenic program (Adi et al., 2002). Finally, ERK inhibition has also been shown to prevent muscle wasting in a C26 colon carcinoma mouse model of cancer cachexia (Penna et al., 2010; Quan-Jun et al., 2016).

Given the promising results of Selumetinib in patients and of ERK inhibition in mice, we sought to investigate Selumetinib in a LLC model of cancer-induced cachexia (Bennani-Baiti and Walsh, 2011). Here we report *in vitro* hypertrophy and *in vivo* tumor killing and inhibition of IL-6 production by Selumetinib in mice, but no evidence of anti-cachexia effects either *in vivo* or *in vitro*.

## Materials and methods

### Cell cultures

Lewis lung carcinoma cells were maintained at low confluence at 37°C in a humidified atmosphere of 5% CO_2_ in DMEM, 10% fetal bovine serum (FBS), 100 U/mL penicillin, and 100 mg/mL streptomycin (pen/strep). Cells were trypsinized, counted and resuspended in PBS for injection.

Murine C2C12 myoblasts (ATCC) were grown in DMEM, 10% FBS and pen/strep. Confluent cells were switched to differentiation medium (DM), consisting of DMEM with 2% horse serum and pen/strep for 96 h. After this time, the medium was replaced with DM containing 10 nM Selumetinib or vehicle for an additional 48 h. For the LLC plasma experiment, C2C12 cells were differentiated for 96 h before being switched to media consisting of DMEM with 2% plasma from control or LLC tumor bearing mice and pen/strep, either with or without 10nM Selumetinib, then incubated for an additional 48 h.

### Animals

All experimental animal protocols were approved by and used in compliance with the Indiana University School of Medicine Institutional Animal Care and Use Committee. Eight-week old male C57BL/6J mice were obtained from The Jackson Laboratory. All mice were maintained on a regular light-dark cycle and allowed free access to food and water throughout the duration of the experiment. Mice were grouped as follows: Control + vehicle (*n* = 6), LLC + vehicle (*n* = 8), and LLC + Selumetinib (*n* = 8). Tumor bearing mice were subcutaneously injected with 10^6^ LLC cells in the intrascapular region on day 0, with treatments beginning 24 h later. Selumetinib (Selleckchem) in vehicle (0.5% methylcellulose/0.2% Tween 80) or vehicle alone was administered twice daily at 25 mg/kg by gavage (Shannon et al., 2009; Troiani et al., 2012; Huang et al., 2013). Body weights of the mice were recorded daily. Mice were euthanized under general anesthesia on day 17 when some mice reached the criteria for a humane endpoint. Muscles, tumors and organs were dissected, weighed, snap frozen in liquid nitrogen, and stored at −80°C. Tissue weights are expressed as a percentage of initial body weight to normalize for small differences in starting size.

### Immunofluorescence and immunohistochemistry

C2C12 cultures were fixed and permeabilized in ice cold acetone/methanol (1:1) at −20°C for 20 min. After 10 min of rehydration in PBS at room temperature (RT), cells were blocked in an 8% BSA solution for 1 h at RT. Primary antibody against myosin heavy chain (Developmental Studies Hybridoma Bank) was incubated overnight at 4°C with gentle agitation. Washed cultures were incubated with AlexaFluor 488-labeled anti-mouse IgG (Life Technologies) for 1 h at RT. Nuclei were stained with DAPI and images were captured on an Axio Observer.Z1 (Zeiss). Myotube diameter was measured using ImageJ analysis software (Wayne Rasband, U.S. National Institutes of Health).

For analysis of muscle fiber cross-sectional area, tibialis anterior muscles were mounted on cork discs with Optimal Cutting Temperature compound, and frozen in 2-methylbutane cooled in liquid nitrogen before being stored at −80°C. Fresh frozen sections were cut using a Leica CM1860 Cryostat (Leica Microsystems Inc.). Muscle sections were fixed in 100% acetone at −20°C before being rehydrated with PBS and blocked in an 8% BSA solution for 1 h at RT. Following overnight incubation with a primary antibody against Dystrophin (Vector Laboratories), sections were incubated for 1 h at RT with an AlexaFluor 594-labeled anti-mouse IgG. Muscle fiber cross-sectional area was measured using an ImageJ macro developed by Dr. Richard Lieber (Minamoto et al., 2007).

Formalin-fixed, paraffin-embedded tumor tissue sections were deparaffinized in xylene and ethanol. Slides were boiled in 10 mM sodium citrate buffer pH 6.0 for 10 min, and cooled at RT for 30 min, then blocked with 8% BSA in PBS for 1 h, followed by overnight incubation at 4°C with antibody against IL-6 (Abcam) or normal rabbit IgG (Santa Cruz Biotechnology). Antibody detection used the ImmPRESS HFP Anti-Rabbit IgG (Peroxidase) Polymer Detection kit and ImmPACT DAB Peroxidase (HRP) Substrate per manufacturer's instructions (Vector Laboratories).

### Western blotting

Muscles and tumor were homogenized on ice in lysis buffer containing 25 nM TrisHCl pH 7.6, 150 mM NaCl, 1% NP-40, 1% sodium deoxycholate, 0.1% SDS, and fresh protease and phosphatase inhibitor cocktail tablets (Roche). Homogenates were centrifuged at 4°C at 14,000 rpm for 15 min, and supernatant was collected and stored at −80°C. Protein concentration was measured by BCA protein assay kit (Thermo Scientific). Protein extracts (30 μg) were denatured at 95°C for 5 min in loading buffer (125 mM Tris pH 6.8, 4% SDS, 20% glycerol, 1% bromphenol blue, and 10% 2-mercaptoethanol). Samples were resolved on Tris-Glycine gels and transferred to nitrocellulose (Bio-Rad Laboratories). Membranes were blocked in SEA BLOCK Blocking Buffer (Thermo Scientific) and incubated overnight at 4°C with antibodies against: IL-6 (EMD Millipore), α-Tubulin (Sigma-Aldrich), Phospho-p44/42 MAPK (ERK1/2) (Thr202/Tyr204), p44/42 MAPK (ERK1/2), and GAPDH (Cell Signaling Technology). Anti-mouse and anti-rabbit IgG conjugated to DyLight 680 and 800 fluorescent dye (Cell Signaling Technology) respectively, were detection antibodies incubated for 1 h at RT. Membranes were imaged and quantified using an ODYSSEY CLx Infrared Imaging System and software (LI-COR).

### IL6 immunoassay

Whole blood was collected at euthanasia, via cardiac puncture, into EDTA tubes (BD Biosciences) and placed on ice. Plasma was separated by centrifugation at 3500 × rpm for 15 min at 4°C and stored at −80°C. IL-6 was detected in duplicate samples by a mouse magnetic 1-plex custom kit as per the manufacturer's instructions (Life Technologies) on a MAGPIX (Luminex).

### Data analysis

For experiments containing only two groups, statistical testing was by unpaired *t*-test. Experiments containing three or more groups, statistical significance was determined by one-way analysis of variance (ANOVA), followed by Tukey's multiple comparisons test. A *p*-value > 0.05 was considered statistically significant.

## Results

### Selumetinib induced C2C12 hypertrophy

**Figure 1:**
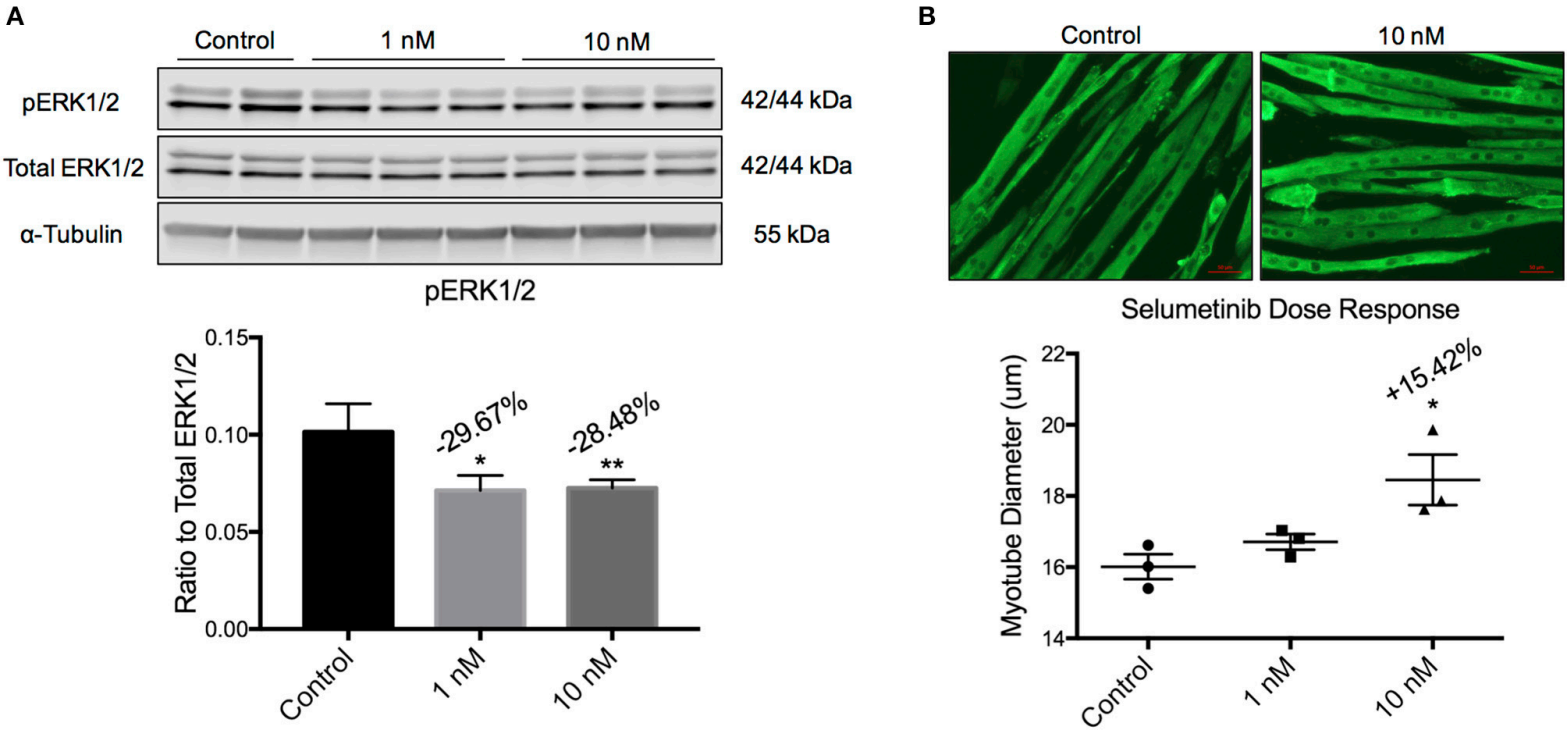
**Selumetinib induces myotube hypertrophy. (A)** Western blotting analysis of C2C12 myotubes shows that 1 and 10 nM of Selumetinib reduced phosphorylated ERK1/2. pERK1/2 was normalized to α-Tubulin. **(B)** Representative myotubes treated with 10 nM Selumetinib or vehicle and stained for myosin heavy chain. Treatment with 10 nM Selumetinib induces myotube hypertrophy (+15.24%). Conditions were performed in triplicate. Data are means ± SEM. ^*^p < 0.05, ^**^p < 0.01 vs. control.

C2C12 myoblasts proliferate as mononuclear cells in growth medium, and are induced to differentiate into syncytial myotubes upon switching to low serum conditions. This system has been used extensively to assess the atrophic or hypertrophic effects of proteins, conditioned medium, serum, or small molecules (Bonetto et al., 2011, 2012). Differentiated C2C12 myotubes were incubated with Selumetinib or vehicle for 48 h, with a media change after the first 24 h. Western blotting showed that 1 and 10 nM Selumetinib reduced ERK1/2 phosphorylation in C2C12 myotubes by ~30% (Figure 1A). Myotube hypertrophy (diameter +15.42%, *P* < 0.05) was observed at a concentration of 10 nM but not 1 nM Selumetinib (Figure 1B), and higher concentrations were toxic (data not shown).

### Selumetinib inhibited ERK1/2 phosphorylation in skeletal muscle

**Figure 2:**
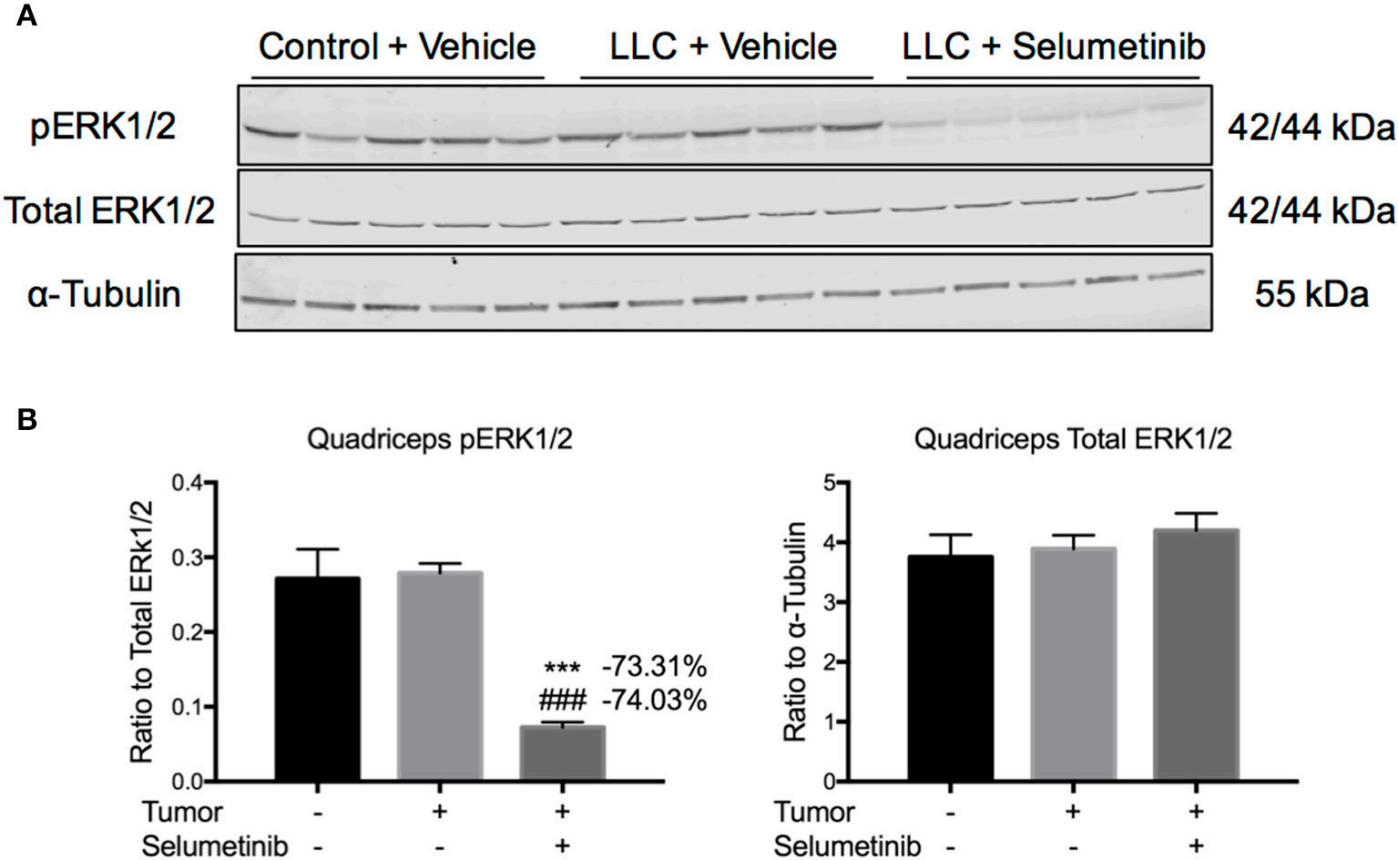
**Selumetinib inhibits ERK phosphorylation in skeletal muscle ***in vivo***. (A)** Western blotting analysis of quadriceps lysates shows reduced pERK1/2 in Selumetinib treated mice, consistent with MEK inhibition. **(B)** Quantification of Western blotting analysis. pERK1/2 was significantly reduced in tumor bearing mice treated with Selumetinib compared to vehicle treated control and tumor bearing mice, while total ERK1/2 was unchanged. Data are expressed as means ± SEM. ^***^p < 0.001 vs. Control + Vehicle, ^*###*^*p* < 0.001 vs. LLC + Vehicle.

To test effects of Selumetinib on tumor growth and body composition in the setting of cancer, we injected mice with LLC cells, a well-validated and traditional model of cancer cachexia. Mice were treated twice daily with 25 mg/kg Selumetinib by gavage. Control mice received PBS injection and vehicle gavage, while tumor-bearing mice received tumor cell injection and vehicle gavage. No differences in overall body weight change or body composition were observed over the course of the experiment (data not shown). Mice were euthanized and necropsied on day 17. To query an on-target effect of Selumetinib in muscle, we performed Western blotting for phospho-ERK1/2. pERK1/2 was decreased 73.31 and 74.03% in the quadriceps of Selumetinib-treated mice vs. vehicle-treated control and tumor-bearing mice respectively (Figures 2A,B, *P* < 0.001). Total ERK1/2 was similar among all groups (Figures 2A,B).

### IL-6 expression decreased in blood and tumor, but not muscle

**Figure 3:**
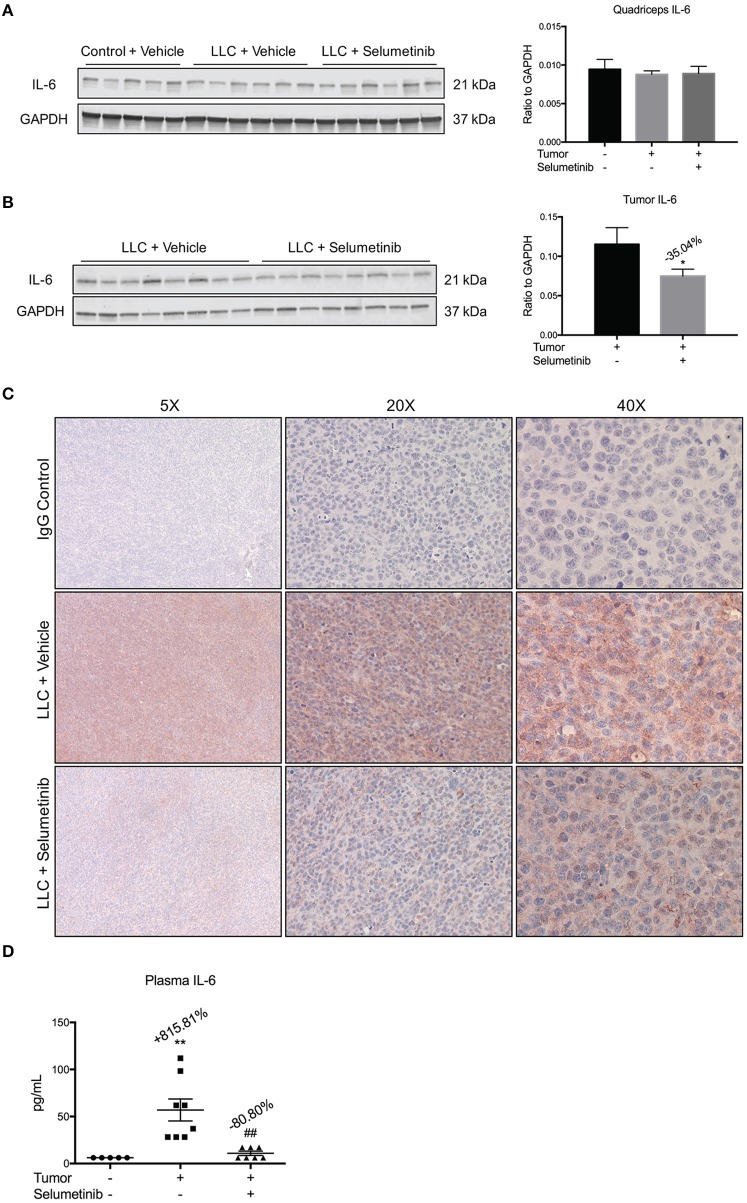
**Selumetinib reduces IL-6 levels in blood and tumor, but not muscle. (A)** Western blotting analysis shows quadriceps IL-6 was not changed in cachexia or with Selumetinib. **(B)** IL-6 expression was reduced in lysates of tumors from mice treated with Selumetinib. Data are expressed as the means ± SEM. **(C)** Representative images of immunohistochemistry. Selumetinib-treated mice show reduced staining for IL-6 in tumor. **(D)** Plasma IL-6 levels were increased in vehicle-treated LLC mice. Selumetinib treated LLC mice showed a significant decrease in circulating IL-6 vs. vehicle-treated LLC mice. Data are expressed as means ± SEM. ^*^p < 0.05 vs. LLC + Vehicle; ^**^p < 0.01 vs. control + Vehicle; ^*##*^*p* < 0.01 vs. LLC + Vehicle.

Given that Selumetinib reportedly blocks production of the pro-cachectic inflammatory cytokine IL-6 (Tai et al., 2007), we measured IL-6 in tissue, tumor and blood. By Western blotting, IL-6 was not decreased in skeletal muscle (Figure 3A). However, Selumetinib decreased IL-6 protein by 35.04% (*P* < 0.05) in tumor lysates (Figure 3B), a finding confirmed by immunohistochemistry of tumor sections (Figure 3C). Furthermore, circulating levels of IL-6 were decreased 80.80% (*P* < 0.01) in Selumetinib-treated LLC mice vs. vehicle-treated tumor bearers (Figure 3D). IL-6 levels in the Selumetinib group were not significantly different from non-tumor bearing mice.

### Selumetinib reduced tumor size, but did not prevent muscle wasting or fat loss

**Figure 4:**
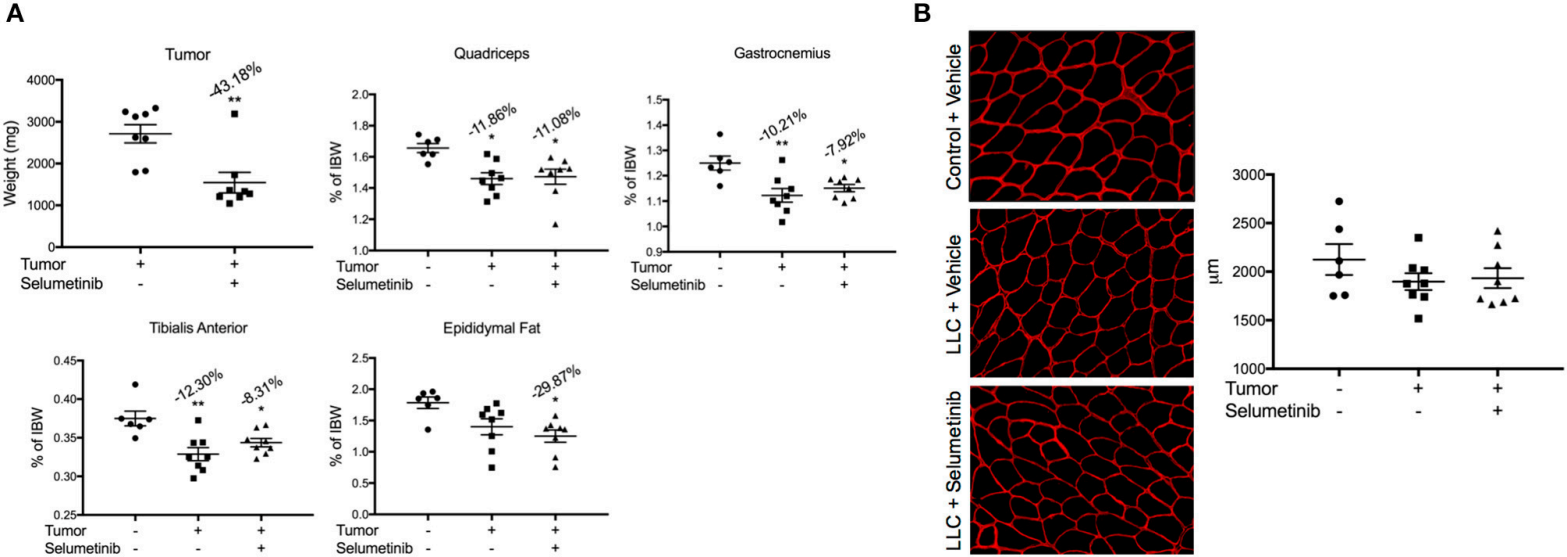
**Selumetinib reduced tumor mass, but did not protect against cancer-induced cachexia. (A)** Tumor mass was significantly reduced in mice treated with Selumetinib. When compared to controls, LLC-bearing mice treated with either vehicle or Selumetinib showed similar wasting in the quadriceps (−11.86 and −11.08%,), gastrocnemius (−10.21 and −7.92%), and tibialis anterior muscles (−12.30 and −8.31%). Epididymal fat loss was significant in the LLC-Selumetinib group. Greater carcass loss was observed in the LLC-Selumetinib mice, while heart loss was greater in vehicle-LLC mice. Data are expressed as means ± SEM. ^*^p = <0.05, ^**^p = <0.01. **(B)** Representative images of muscle fiber cross-sectional area and quantification. Data are expressed as means ± SEM.

Consistent with its anti-tumor effects in other models of non-small cell lung cancer, Selumetinib treatment reduced LLC tumor size by 43.18% (*P* < 0.01) (Figure 4). Given that tumor size was greatly reduced, we expected muscle wasting to be attenuated, because in this model severity of cachexia generally correlates with tumor burden. However, in all muscles analyzed, both the Selumetinib and vehicle-treated LLC mice showed similar wasting (Figure 4A). Analysis of muscle fiber cross-sectional area displayed the same pattern as those observed in the muscle weights (Figure 4B). Greater fat loss and carcass loss were observed in Selumetinib-treated mice (Figure 4A).

### Selumetinib did not prevent LLC plasma-induced C2C12 myotube atrophy

**Figure 5:**
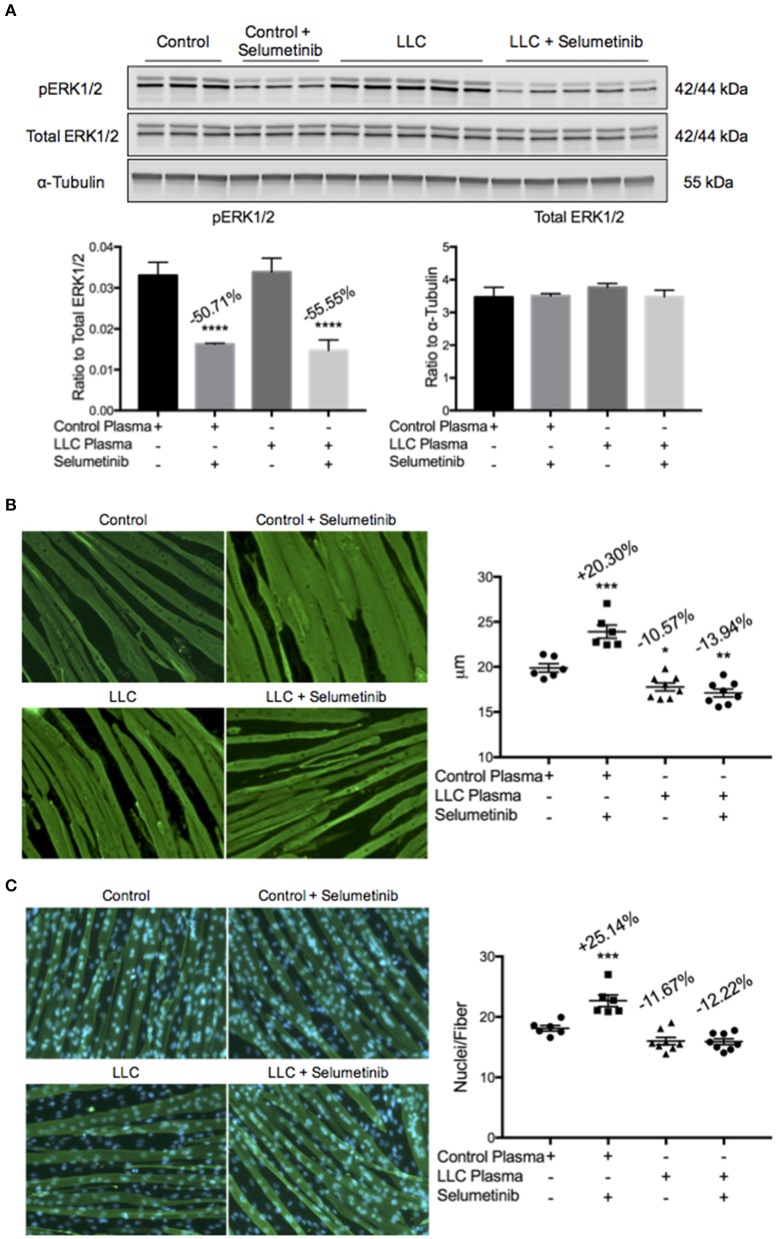
**ERK inhibition does not block LLC plasma-induced atrophy**. C2C12 myotubes were treated with control plasma, control plasma + 10 nM Selumetinib, LLC plasma, or LLC plasma + 10 nM Selumetinib. Each data point represents myotubes treated with plasma from one individual mouse. **(A)** Western blotting analysis of C2C12 myotubes incubated with control (−50.71%) or LLC plasma (−55.55%) show inhibition of ERK1/2 phosphorylation upon treatment with Selumetinib. Data are expressed as means ± SEM. ^****^p = <0.0001. **(B)** Representative images and quantification of myotube diameter of C2C12 cells. Myotubes incubated with control plasma and treated with Selumetinib showed significant hypertrophy (+20.30%) vs. the control plasma only group. Both the LLC plasma only (−10.57%) and the LLC plasma with Selumetinib (−13.94%) groups showed atrophy vs. control plasma only. Data are expressed as means ± SEM. ^*^p = <0.05, ^**^p = <0.01, ^***^p = <0.001. **(C)** Representative images and quantification of nuclei per fiber. The total number of fibers counted were the same amongst all groups. Selumetinib treatment increased nuclei per fiber (+25.14%) when compared to control plasma only. LLC plasma showed a decrease (−11.67%) vs. control plasma only, which Selumetinib treatment was unable to block (−12.22%), although these were not statistically significant. Data are expressed as means ± SEM. ^***^p = <0.001.

While a reduced tumor burden and decreased circulating levels of IL-6 were seen with Selumetinib treatment, we did not observe any protection in skeletal muscle or fat mass. This led us to question whether there were other cachexia drivers in the LLC model that Selumetinib treatment could not modulate. To explore this, we treated C2C12 myotubes with plasma from either control mice or vehicle treated tumor bearing mice. This was done both with and without 10 nM of Selumetinib, the concentration previously used to induce myotube hypertrophy. Western blotting analysis showed that Selumetinib was able to reduce expression of pERK1/2 50.71 and 55.55% in myotubes incubated with control or LLC plasma, respectively (Figure 5A). Consistent with our prior *in vitro* data (Figure 1), Selumetinib treatment was able to induce significant hypertrophy in myotubes incubated with control plasma. However, similar to our *in vivo* results, Selumetinib was unable to block myotube wasting induced by LLC plasma (Figure 5B). Further analysis showed that in addition to increasing myotube diameter, ERK inhibition also increased the number of nuclei per fiber (Figure 5C). Myotubes treated with LLC plasma showed a reduction in the number of nuclei per fiber, although this was not statistically significant, which Selumetinib treatment was again unable to attenuate.

## Discussion

Here we show that unlike results reported in patients with biliary cancers, mice with lung cancer do not exhibit reduced lean muscle loss despite tumor response with Selumetinib. This result was surprising for three reasons. Firstly, Selumetinib increased C2C12 fiber size *in vitro*, suggesting a potential pro-anabolic effect in skeletal muscle. In addition, we observed an increase in the number of nuclei per fiber with Selumetinib, suggesting that ERK inhibition increased the differentiation or fusion potential of myoblasts. However, despite reducing pERK1/2 in skeletal muscle, Selumetinib did not result in muscle protection much less hypertrophy in LLC conditions. Secondly, Selumetinib significantly inhibited tumor growth. Tumor mass normally correlates with the severity of muscle wasting, thus reduction of tumor burden should have led secondarily to reduced cachexia. This disconnect between tumor size and cachexia suggests that Selumetinib actually enhanced pro-cachectic pathways in LLC mice. Thirdly, those pathways must also be independent of IL-6, given that circulating and tumor-derived IL-6 were reduced in our study. This conclusion is supported by the observation that Selumetinib was unable to block LLC plasma-induced myotube atrophy. These data suggest that another, or several other, inflammatory cytokines or circulating factors are the essential driver/s of muscle wasting in the LLC model, not IL-6.

It is possible that the effects of Selumetinib on tumor growth and muscle wasting are disease specific, because Selumetinib was associated with increased lean body mass in patients with biliary cancers (Prado et al., 2012) and in the murine C26 colon adenoma cachexia model. Biliary, colon and lung cancers might exert muscle wasting through different effectors. In the C26 studies, ERK inhibition had no effect on tumor mass in one study (Penna et al., 2010), but resulted in an ~15% decrease in tumor mass in another study (Quan-Jun et al., 2016). However, the studies each used different inhibitors and the mice from both were of a different genetic background than those used here.

The MEK pathway might also play different roles in humans vs. murine cancer cachexia. In the phase II clinical trial, 52% of patients treated with Selumetinib experienced a decrease in target lesion size, similar to what we observed in the present study. This could potentially explain the gain in total body mass of patients treated with Selumetinib, as opposed to the loss in patients receiving standard therapy. The increased muscle mass could be a result of a reduced tumor burden, and not any direct effect on the skeletal muscle itself. The authors hypothesize that the anabolic effect of Selumetinib is likely attributed to the inhibition of cytokine secretion. However, here we observed a significant decrease in both tumor tissue and circulating levels of IL-6, but with no beneficial effects on skeletal muscle.

Finally, it is possible that the lack of muscle preservation is due to the differential regulation and requirements of the MEK pathway during myogenesis. While we did not investigate muscle satellite cells in this study, it is possible that constant inhibition of the pathway led to a defect in proliferation or depletion of the satellite cell pool. Literature shows that ERK signaling can be both stimulatory and inhibitory for muscle differentiation. ERK1/2 activation is necessary for satellite cell proliferation and self-renewal (Ogura et al., 2015; Hindi and Kumar, 2016), but not required for fusion or expression of muscle specific genes (Jones et al., 2001). In addition, ERK2 is necessary for myotube formation, as siRNA-mediated knockdown of ERK2 in C2C12 myoblasts inhibited their fusion into multinucleated myotubes (Li and Johnson, 2006). Akt activation, a positive regulator of muscle mass, leads to inhibition of the MEK pathway in differentiated myotubes, while having no effect on their muscle precursor cells (Rommel et al., 1999). Conversely, leukemia inhibitory factor, an IL-6 family cytokine, inhibits myogenic differentiation through phosphorylation and activation of ERK1/2 (Jo et al., 2005). *In vitro* data show that early ERK1/2 activation, within 24 h post differentiation induction, can repress myogenic differentiation. Inhibition of MEK1 in the latter stages of differentiation displayed similar effects, blocking myotube formation (Jo et al., 2009). These data suggest that myogenic differentiation is coordinated by low MEK1 activity during the initial phases, and high activity thereafter. As such, while constant administration of Selumetinib inhibits tumor growth, achieving an anabolic effect appears to be more complicated.

Due to the requirements for ERK1/2 modulation in myogenesis, constant inhibition of ERK1/2 may be detrimental to skeletal muscle mass. The studies mentioned were able to control the myogenic stages at which the pathway was perturbed. While this would be challenging to accomplish *in vivo*, a potential approach would be to treat intermittently. This approach would allow for pathway activation, instead of remaining under a constant state of inhibition. Based upon the literature, allowing for cycles of activation and inhibition could potentially produce the stimulatory effects necessary for muscle hypertrophy. Future investigation will be necessary to determine a proper dosing regimen in order to determine the therapeutic potential of ERK inhibition as a treatment for cachexia and the potential effects of such cyclic dosing on tumor growth.

Taken together, these data suggest the need to consider the differential regulation of not only the MEK and IL-6 pathways, but also other pathways in muscle wasting of cancer cachexia. Moreover, they point to profoundly different drug-responsive phenotypes in commonly used cachexia models, suggesting diversity in the underlying cellular and molecular mechanisms and the need for care in extrapolating results across disease states, clinical trials and model systems.

## Author contributions

EA and AD carried out experiments, collected and interpreted data. LK and TZ designed and directed experiments. EA and TZ wrote the manuscript. TZ obtained funding for the studies.

### Conflict of interest statement

The authors declare that the research was conducted in the absence of any commercial or financial relationships that could be construed as a potential conflict of interest.
